# The treatment of acute promyelocytic leukemia in 2023: Paradigm, advances, and future directions

**DOI:** 10.3389/fonc.2022.1062524

**Published:** 2023-01-18

**Authors:** Sunil Girish Iyer, Laila Elias, Michele Stanchina, Justin Watts

**Affiliations:** ^1^ Department of Medicine, Division of Hematology, University of Miami Miller School of Medicine, Miami, FL, United States; ^2^ Sylvester Comprehensive Cancer Center, University of Miami Miller School of Medicine, Miami, FL, United States; ^3^ University of Miami Miller School of Medicine, Miami, FL, United States

**Keywords:** acute promyelocytic leukemia, all-trans retinoic acid (ATRA), arsenic trioxide (ATO), differentiation syndrome, disseminated intravascular coagulation (DIC), coagulopathy, oral arsenic

## Abstract

The transformation of acute promyelocytic leukemia (APL) from an often fatal to highly curable cancer with long-term survival exceeding 90% is one of the greatest and most inspiring successes in oncology. A deeper understanding of the pathogenesis of APL heralded the introduction of highly effective therapies targeting the mutant protein that drives the disease, leading to the chemotherapy-free approach to cure almost all patients. In this review, we discuss the paradigm of treatment of APL in 2023, reinforce the high risk of early death without prompt initiation of treatment at first clinical suspicion, and dedicate a special focus to novel agents and future directions to improve cure rates and quality of life in patients affected by APL.

## Introduction

Acute promyelocytic leukemia (APL) is a unique subtype of acute myeloid leukemia (AML), accounting for about 15% of AML cases (1). In comparison to other morphological subtypes of AML, APL is a distinct clinical entity characterized by a marked tendency towards coagulopathy, hemorrhage, and early death (2); however, with modern therapies, APL has become the most curable acute leukemia with long-term disease-free survival (DFS) approaching 90% (3–7). APL is defined by the reciprocal translocation t (15;17), involving the promyelocytic leukemia (PML) gene on chromosome 15 and the retinoic acid receptor alpha (RARα) gene on chromosome 17 (8). This translocation begets the formation of the PML-RARα fusion gene, which acts as a transcriptional repressor inhibiting normal myeloid differentiation. The myeloid precursors harboring this cytogenetic aberration remain arrested in the promyelocytic stage, accumulating in the bone marrow and the blood (9, 10). Patients with AML typically present with symptoms related to the suppression of normal bone marrow function such as fatigue, infection, and bleeding. Uniquely, APL often presents with an aggressive coagulopathy related to disseminated intravascular coagulation (DIC) with primary hyperfibrinolysis (2, 11, 12).

**Figure 1:**
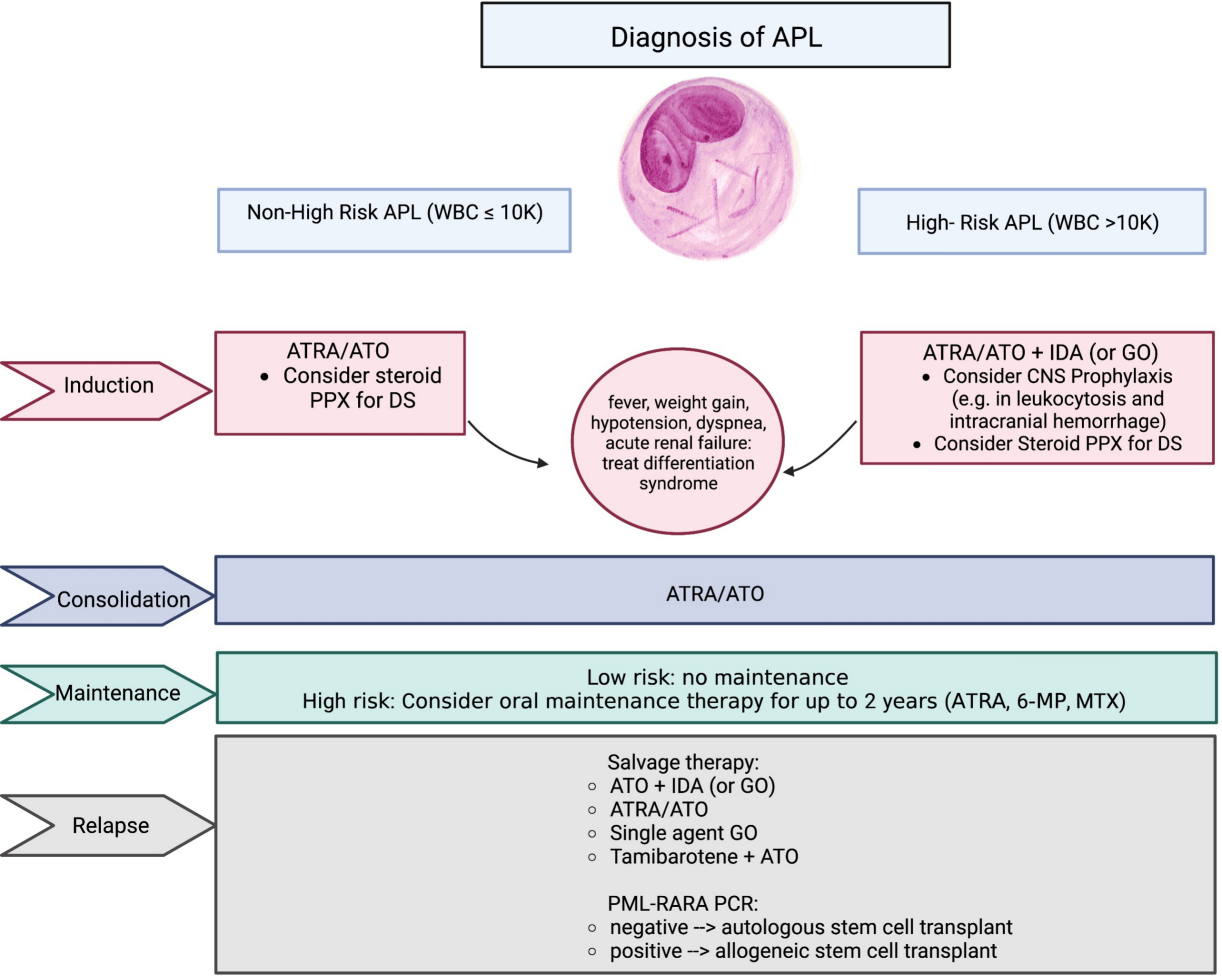
Summary of key agents used in the treatment of APL. APL, acute promyelocytic leukemia; WBC, white blood cell count; ATRA, all-trans retinoic acid; ATO, arsenic trioxide; GO, gemtuzumab ozogamicin; PPX, prophylaxis; DS, differentiation syndrome; 6-MP, 6-mercaptopurine; MTX, methotrexate; PCR, polymerase chain reaction.

Historically, APL was treated with standard AML-directed chemotherapeutic induction regimens, with high rates of relapse and early death (13–15). In the past decades, therapies targeting the specific genetic aberration directly implicated in the pathogenesis of APL have transformed the prognosis of this disease and has offered a chemotherapy-free approach for definitive curative therapy. In this perspective, we discuss the modern paradigm of the management of APL during all phases of treatment (induction, consolidation, and maintenance), as well as emerging innovations and future directions of treatment such as oral formulations of arsenic trioxide (ATO). Key agents discussed in this review are summarized in **Figure 1**.

### Evolution of the induction paradigm

Prior to the introduction of all-trans retinoic acid (ATRA) into the treatment armamentarium, APL induction consisted of standard AML-directed intensive chemotherapeutic regimens containing an anthracycline and cytarabine. Initial remission rates were favorable within the spectrum of AML at 70-80%; however, over half of responding patients eventually relapsed and the 2-year overall survival (OS) was 30-40% (13, 14). In 1988, groundbreaking data from a Chinese group demonstrated that ATRA could induce differentiation of promyelocytic blasts and yield morphological remission in 100% of treated patients, some of whom were refractory to chemotherapy, without bone marrow hypoplasia (16). Subsequent studies in the early 1990s confirmed the efficacy of ATRA in inducing differentiation of the leukemic clone; however, there was a high incidence of early death due to coagulopathy and what is now known as differentiation syndrome (DS), as well as high relapse rates (17–19).

In an effort to decrease the incidence of relapse, ATRA was combined with anthracycline-containing chemotherapy in a sequential manner and showed superiority in the reduction of relapse rates versus chemotherapy alone (20). The “AIDA” regimen combined ATRA and idarubicin in a concurrent manner, moving the anthracycline to the first week of induction with the omission of cytarabine, and showed reduced rates of DS, thus becoming the standard of care (21–23). Sequential and concurrent dosing of ATRA and chemotherapy were compared head-to-head in a study published in 1999, demonstrating a relapse rate of 6% at two years in the concurrent group vs 16% in the sequential group, further supporting the early incorporation of chemotherapy (24).

In 2013, Lo-Coco et al. showed that combining ATO with ATRA in patients with non-high-risk APL led to an astounding 2-year DFS of 97% vs 86% in the ATRA plus idarubicin arm with less toxicity, again shifting the paradigm of APL treatment. Consolidation consisted of further treatment with ATRA and ATO, and maintenance therapy was not employed. Long-term follow up at 72 months showed persistent event free survival (EFS) at 96.6% in the ATRA/ATO group versus 77.4% in the ATRA/chemotherapy group (25). Of note, patients may experience delayed count recovery with this approach as the APL clone terminally differentiates, and a positive PML-RARA PCR at count recovery does not necessarily portend resistant disease (5). We typically administer at least 28 days of ATRA/ATO if counts recover quickly but no more than 35 days if recovery is delayed.

### Risk stratification

In the era of ATRA, APL has been stratified into three categories of risk with regards to relapse-free survival (RFS): WBC >10,000/μl (high-risk), WBC ≤10,000 μl with platelet count ≤40,000/μl (intermediate-risk), and WBC ≤10,000/μl with platelet count >40,000/μl (low-risk) (26, 27). Today, most clinicians will categorize APL within two categories, high risk (>10,000/μl) and low/intermediate risk. Patients with leukocytosis on presentation are known to experience a higher rate of early death and life-threatening complications before or during induction, with differentiation of promyelocytic blasts to neutrophils resulting in a systemic inflammatory and vasoactive response and with cytolysis of malignant cells thought to cause release of procoagulant factors (28). The incorporation of chemotherapy into induction for patients with WBC >10,000/μl for rapid cytoreduction is thought to reduce early death and improve outcomes. Patients with high-risk APL, especially those who experience intracranial hemorrhage, are at risk of leukemic relapse in the central nervous system (CNS), which acts as a sanctuary site from systemic therapy. Although ATRA and ATO do penetrate the CNS, studies differ on whether therapeutic levels are reached (15, 29–31), and the use of upfront cytotoxic agents is thought to reduce the risk of CNS infiltration. The APML4 study in 2015 studied an induction strategy utilizing ATRA, ATO, and idarubicin, with an improved 5-year disease-free survival of 83% and OS of 87% in high-risk patients (7), further establishing ATRA/ATO/chemotherapy as the standard of care in high-risk disease.

### Chemotherapy-free induction

With the success of chemotherapy-free induction in non-high-risk APL resulting in a 50-month DFS of over 97% in the APL0406 trial (6), anthracycline-sparing induction has been explored in high-risk disease in an attempt to mitigate cardiac and hematological toxicity and reduce requirements for supportive care. In 2010 a study from MDACC showed that the addition of gemtuzumab ozogamicin (GO), an anti-CD33 antibody conjugated to the cytotoxic agent calicheamicin, to ATRA with ATO produced a complete response (CR) rate of 98% and a 5-year EFS rate of 86% (32). Next, in 2015, an arm of the AML17 study conducted in the United Kingdom showed that ATRA plus ATO (at higher doses over fewer days than in the Lo-Coco and APML4 protocols) combined with GO produced similar CR and overall response rates (ORR) as compared to ATRA/ATO/idarubicin, with no cases of treatment-related AML as opposed to one case in the chemotherapy-containing arm in the UK study (33). A phase 2 study in 2020 confirmed the efficacy of ATRA with ATO and GO in the treatment of high-risk APL; however, post-remission therapy was intensive and included further rounds of GO as well as daunorubicin, and over a third of patients were unable to complete all phases as planned (34).

**Figure 2:**
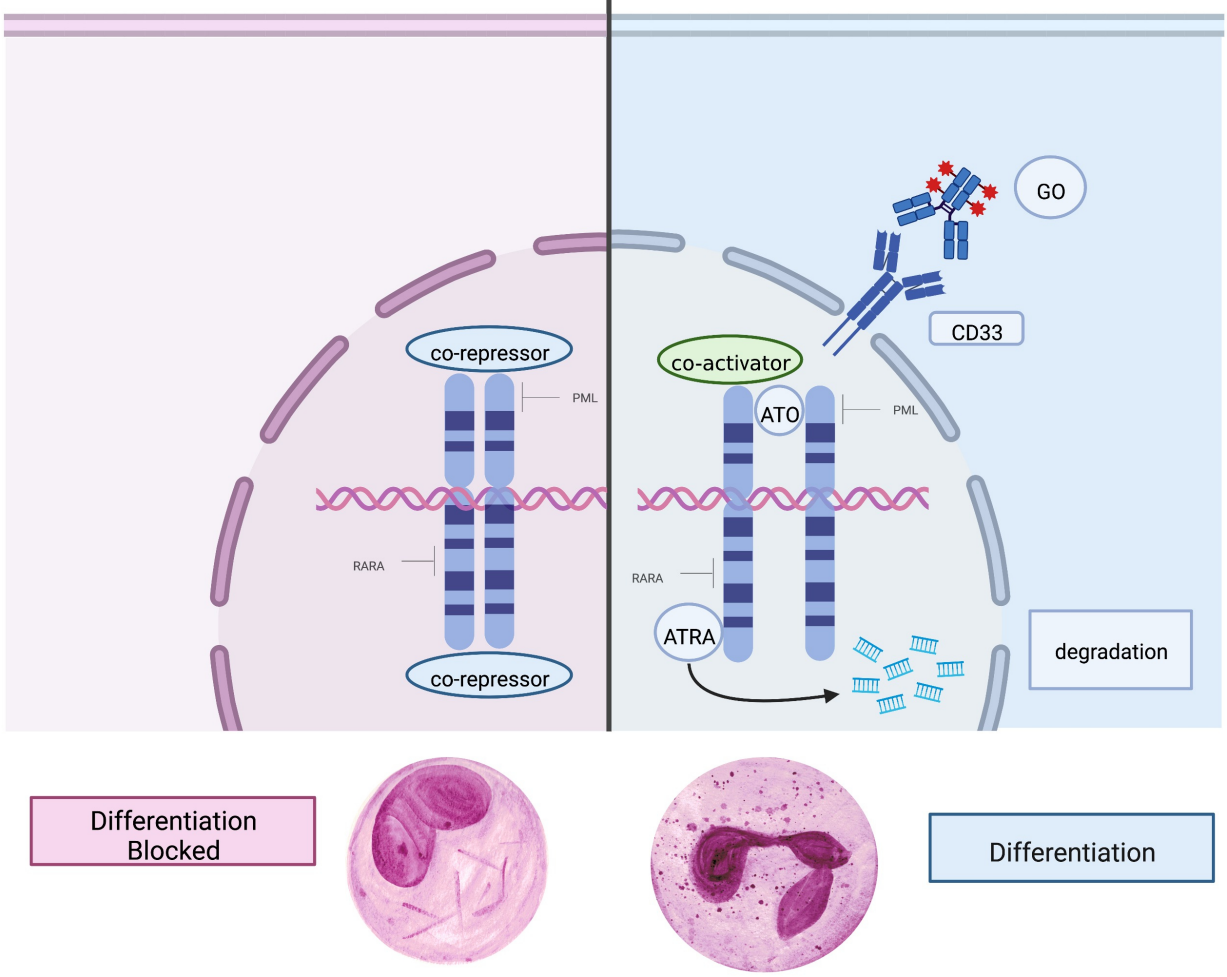
Mechanism of action of key agents. Left: In APL, the PML-RARα fusion protein causes a block in differentiation and drives malignant promyelocytic proliferation. Right: The mutated retinoic acid receptor on the cell surface is targeted and bound by ATRA (all-trans retinoic acid) and ATO (arsenic trioxide), which promotes terminal differentiation, degradation of oncogenic proteins, and apoptosis. GO (gemtuzumab ozogamicin) is an antibody-drug conjugate which binds CD33 on the cell surface and when endocytosed releases the chemotherapeutic agent calicheamicin, causing cell death.

The mechanism of action of ATRA, ATO, and GO are stylized in **Figure 2**. Although ATRA with ATO and an anthracycline is the preferred induction regimen for patients with high-risk APL at most centers, GO remains an alternative to anthracycline, especially for patients ineligible for anthracycline therapy due to cardiac and other comorbidities; this is reflected in the current NCCN guidelines (35).

The clinical question remains whether high-risk patients can be induced with ATRA and ATO, without chemotherapy. In the 2015 AML17 study, patients in all risk groups were randomized to induction with either ATRA with idarubicin or ATRA with ATO; however, the protocol allowed the option to give a dose of GO to high-risk patients. Consequently, GO was given in all high-risk cases in which GO was available, making an analysis of ATRA with ATO only in high-risk patients impossible in this study (33). A phase III study in China has evaluated the use of ATRA and ATO with and without chemotherapy in all risk groups, with the addition of mannitol in high-risk disease with the intention of improving the CNS penetration of ATO; although the results are currently under peer review and not yet published, preprinted results report no significant difference in two-year DFS in high-risk APL between the chemotherapy and non-chemotherapy arms without any occurrences of CNS disease (36). In addition, aggressive use of hydroxyurea may also be sufficient to allow for a chemotherapy-free approach even in high-risk patients (37). Hydroxyurea should be used in appropriately high doses in low-risk patients who have ATRA-induced leukocytosis, along with best supportive care and platelet transfusion support. Lastly, we recommend the prophylactic administration of intrathecal chemotherapy for patients with WBC >10,000/μl at diagnosis or prior CNS hemorrhage, delivered at the end of the first cycle when blood counts have recovered; Isolated CNS relapse, although infrequent, can occur and has been associated with these risk factors (38–40).

### Additional cytogenetic and molecular aberrations

In addition to PML-RARA, there are 16 additional known RARA rearrangements with alternative fusion partners; these rearrangements are collectively known as variant APL. Promyelocytic leukemia zinc finger (PLZF), also known as ZBTB16, is a transcriptional repressor involved in cell cycle control and myeloid cell differentiation. PLZF is located on chromosome 11q23; t(11;17)(q23;q21) generates PLZF-RARA rearrangements, accounting for about 1% of APL. APL with PLZF-RARA rearrangements is more resistant to treatment with ATRA and ATO (41). The second most common variant is NPM1-RARA. NPM1 is a nucleolar phosphoprotein located on chromosome 5q32 and is involved in molecular chaperoning, ribosome synthesis, DNA repair, and genome stability. Of note, NPM1-RARA has also been identified in atypical acute myelomonocytic leukemia, chronic myeloid leukemia, cutaneous mastocytosis, and myeloid sarcoma, and can activate multiple signaling pathways. Unlike APL with PLZF-RARA, APL with NPM1-RARA appears to be relatively ATRA-sensitive (41, 42). Other variant RARA rearrangements include NUMA-RARA, STAT5B-RARA, PRKAR1A-RARA, BCOR-RARA, FIP1L1-RARA, among others, and their clinical significance continues to be explored (42).

RARB rearrangements on chromosome 3p24 can also cause variant APL, specifically the TBL1XR1-RARB rearrangement. APL with RARB rearrangement is ATRA-resistant and while treatment with combined chemotherapy has shown to be more effective, most of the patients will relapse. Similarly, RARG rearrangements, including NUP98-RARG, PML-RARG, CPSF6-RARG, NPM1-RARG-NPM1, and HNRNPC-RARG, have been identified and also show resistance to standard ATRA-based therapy. Very rarely, morphological and immunophenotypic APL with non-RAR rearrangements can occur involving fusion of MLL with partners such as ELL, AF1Q, and RPRD2; only three such cases have been reported, with successful induction with AML-directed intensive chemotherapy and discontinuation of ATRA as ATRA-sensitive RAR mutations were not detected (43, 44). Overall, atypical variants of APL are important to identify by cytogenetic analysis, as they may be resistant to conventional APL-directed therapy and may require modification of treatment (42).

Somatic alterations in multiple genes, including *fms-like tyrosine kinase 3 (FLT3)* and NRAS, are frequently detected by next-generation sequencing in patients with APL. It is unknown how these mutations cooperate with PML-RARA rearrangement to induce or propagate leukemogenesis, and in the era of ATRA/ATO-based therapy there is no singular mutation known to confer any prognostic impact, positive or negative (45, 46). Activating mutations in FLT3, which can occur as tandem duplication (ITD) or tyrosine kinase domain (TKD) mutations, are associated with increased tumor burden and risk of relapse in non-APL AML and are considered to be a negative prognostic marker (47). These mutations occur frequently in APL, occurring in 35-40% of cases as opposed to in 20-25% of non-APL AML cases. A recent study in a murine model demonstrated that the presence of FLT3-ITD confers resistance to ATRA-mediated degradation of PML-RARA; however, treatment efficacy is salvaged by concurrent treatment with ATO, effectively circumventing resistance (48). Indeed, a retrospective analysis of patients with APL treated with ATRA and chemotherapy showed worsened survival in the presence of FLT3 mutation with a variant allele fraction (VAF) >50% (49). A more recent analysis performed in the era of ATO did not show a prognostic influence of FLT3 mutation in patients with APL treated with ATRA/ATO (50).

### Management and prevention of differentiation syndrome

Retinoic acid (RA) is integral to regulating differentiation pathways such as myelopoiesis. During normal cell differentiation, RA binds to its corresponding receptor, inducing a conformational change that releases corepressors and induces co-activators to active transcription. The PML-RARα oncoprotein represses RA signaling, interfering with terminal myeloid differentiation, leading to maturation arrest at the promyelocyte stage (51).

Treatment with ATRA, which targets the RA receptor and induces terminal differentiation of APL blasts, and ATO, which degrades the PML-RARα fusion protein and induces apoptosis, produce deep clinical responses, but are associated with DS, also known as the retinoic acid syndrome (RAS). DS is a relatively common yet potentially life-threatening side effect of induction therapy characterized by fever, hypotension, renal failure, and fluid retention which can manifest as pleural and pericardial effusions, peripheral edema and weight gain, and respiratory distress with pulmonary infiltrates. It has been proposed that the pathogenesis of DS is mechanistically like that of systemic inflammatory response system (SIRS). As ATRA drives APL blasts into maturation, pro-inflammatory cytokines such as interleukin (IL)-1, IL-β, IL-6, IL-8, and tumor necrosis factor α, are released which can lead to fever, tachypnea, and tachycardia, leading to cardiopulmonary shock if left untreated. Additionally, the release of cathepsin G causes endothelial damage and increases vascular permeability, inducing capillary leak syndrome which can lead to tissue hypoperfusion and end organ damage. Induction therapy may also increase chemotaxis of promyelocytes and blasts as they undergo rapid differentiation to neutrophils, leading to increased adhesion of blasts and leukocytosis, further causing endothelial damage and precipitating organ failure (15, 52, 53)

Diagnosing DS in a timely manner is integral to reducing mortality during induction for APL, especially in high-risk APL patients. Early diagnosis remains challenging as signs and symptoms are non-specific and can be confounded by other concurrent medical conditions. While it is crucial to rule out confounding conditions such as heart failure, pneumonia, infection, pulmonary embolus, diffuse alveolar hemorrhage, and other causes of renal failure, it is imperative to keep a high index of suspicion for DS and remain vigilant about monitoring labs, vital signs, and daily weights. Several groups have advocated for the use of prophylactic corticosteroids either with prednisone 0.5mg/kg per day on day 1 until the end of induction, prednisone 1mg/kg per day on Days 1-10, or dexamethasone 2.5mg/m2 every 12 hours on days 1-15, although there is no consensus on exact dosing and timing (52, 53). Dexamethasone 10 mg every 12 hours should be administered at the first sign or suspicion of DS for a minimum of three days or until signs and symptoms have resolved (52–54). If DS does not improve, dexamethasone can be increased to 10mg every 6 hours; however, if the patient continues to decompensate, exhibiting severe organ dysfunction or requiring admission to the intensive care unit (ICU), ATRA and ATO must be temporarily discontinued until resolution of organ dysfunction (53). Aggressive cytoreduction with hydroxyurea and/or idarubicin should also be used with close monitoring and correction of the coagulopathy.

### Management of coagulopathy

The bleeding diathesis inherent to APL, which can manifest along a spectrum from minor bruising to life-threatening DIC and hemorrhage, is often responsible for the presenting features of the disease and is the major cause of early mortality. Although prompt initiation of ATRA can reverse this coagulopathy, early death from bleeding within the first 30 days of presentation remains a major cause of treatment failure (15, 55). Cooperative groups have reported a 5-10% early death rate (EDR) in the first month of therapy; however, this number may represent an underestimation of early mortality as many these patients can die prior to official diagnosis or transfer to a treatment center (22, 24, 55). Recent retrospective studies have a reported even higher EDR: In 70 patients presenting to Stanford Hospital with newly diagnosed APL, the EDR was 26% (56). The Swedish Adult Acute Leukemia Registry reported an EDR of 29% in 105 patients with APL (57). A study using the population-based Surveillance, Epidemiology, and End Results (SEER) database reported an EDR of 17.3% among 1400 patients; Additionally, the EDR does not appear to have changed significantly despite routine use of ATRA (58). This may be due to delayed diagnosis, delayed administration of ATRA, or inadequate supportive care.

Almost all cases of fatal hemorrhage occur in the first month of APL therapy, with over half occurring within the first week of treatment (57–59). Rapid initiation of ATRA should begin at first suspicion of APL and should not be delayed for bone marrow aspiration or specialist consultation. DIC can provoke life-threatening bleeding due to thrombocytopenia, depletion of coagulation factors, and disruption of normal fibrin polymerization and platelet aggregation (60). ATRA can mitigate this process by interfering with the expression of procoagulant, fibrinolytic, and proteolytic processes, and by attenuating the secretion of inflammatory cytokines (61). Rapid resolution of bleeding symptoms has been noted in patients promptly treated with ATRA, and laboratory studies have confirmed the decrease or normalization of clotting and fibrinolytic variables within the first few weeks of therapy (61–64). In addition to ATRA administration, coagulopathy should be supported through transfusion with cryoprecipitate or plasma to maintain the fibrinogen concentration above 100-150 mg/dL, and platelet transfusions to increase the platelet count to more than 30,000-50,000/μl. Fresh frozen plasma (FFP) can be administered if the prothrombin (PT) or activated partial thromboplastin time (aPTT) are prolonged (15, 23, 54, 65).

Awareness of emergency room physicians and internists about the rapid administration of ATRA at first suspicion of APL is critical to reducing early mortality. A SEER registry study identified that out of the 23 APL treatment centers in Michigan and Louisiana, only six had ATRA available (66). This highlights the importance of raising general awareness of EDR among APL patients and improving access to ATRA in hospitals across the United States. Additionally, it has been noted that the EDR at community hospitals without rapid access to specialty care is high (67). Furthermore, there is minimal to no harm if ATRA is given for misdiagnosed APL, but a great potential benefit if diagnosis is confirmed. In summary, current guideline recommendations establish that APL should be managed as a medical emergency upon first suspicion, with immediate therapeutic actions aimed at counteracting the coagulopathy with prompt administration of ATRA.

### Consolidation

Although patients may achieve hematologic normalization and molecular clearance of PML-RARA by the end of induction, consolidation therapy remains essential to prevent relapse (68). Cumulative anthracycline exposure poses risks of excess hematologic and cardiac toxicity, and efforts have been made to discern which patients can be effectively consolidated without chemotherapy. Sanz and colleagues introduced a risk stratification discussed above, with a risk-adapted approached serving as the basis for today’s standard of care (27). Following the introduction of ATO, a 2010 study found an advantage in DFS in all risk with the addition of ATO to ATRA, cytarabine, and daunorubicin-containing induction and consolidation (69). With the benefit of ATO in consolidation proven, subsequent studies examined the omission of chemotherapy. A 2010 trial explored induction with ATRA and ATO (with GO added for high-risk patients) with post-remission therapy consisting of ATRA and ATO only; 3-year OS was 85% (32). An Indian group explored induction and consolidation with single-agent ATO; outcomes were comparable to historical controls in low/intermediate risk patients, but relapse rates were higher in high-risk patients than seen historically with chemotherapy-containing therapy (70).

It is now known that low/intermediate-risk patients achieve excellent outcomes with chemotherapy-free ATRA/ATO-based consolidation following ATRA/ATO-based induction (5), and that high-risk patients who are successfully induced with ATRA/ATO with an anthracycline can be consolidated with ATRA/ATO with the omission of chemotherapy (7). With the widespread adoption of ATO, it is unclear if there remains any benefit of cytarabine exposure in high-risk patients, unless their CNS is positive for disease at diagnosis (71).

### Maintenance

There remains unanswered the fundamental question as to which subset of patients will derive benefit from maintenance therapy, and which patients may derive harm from overtreatment of a disease that has already been cured. The risk of harm from chemotherapy-containing maintenance therapy is certainly real, as seen by the increase in death in CR in elderly patients receiving maintenance in a large European study (24). On the other hand, large studies have shown a benefit from maintenance therapy in patients with high-risk disease (70). Maintenance therapy including ATRA, methotrexate, and 6-mercaptopurine (6-MP) is included for high-risk patients in the widely utilized APML4 protocol and omitted for low/intermediate-risk patients (7). For patients receiving protocols utilizing ATO in both induction and consolidation, such as APML4, some groups recommend the omission of maintenance for all risk groups (54). Maintenance is generally omitted in low/intermediate-risk patients having received ATO in induction and consolidation.

Monitoring of measurable residual disease (MRD) by measurement of the PML-RARα fusion protein by polymerase chain reaction (PCR) has been examined to guide whether patients should receive maintenance. In a North American study, low/intermediate risk patients who received induction with ATO and intensive chemotherapy and were MRD-negative by the end of induction did not derive any benefit from maintenance, as there were no relapses in neither the group that was randomized to maintenance nor the group which proceeded to observation only following consolidation (72). The AIDA 0493 protocol, in which patients were induced with ATRA and idarubicin and consolidated with chemotherapy, mandated that patients in remission following consolidation be randomized to four maintenance groups: 6-MP and methotrexate, ATRA alone, ATRA alternating with 6-MP and methotrexate, and no maintenance. There was no difference in 12-year DFS detected among the four groups. A retrospective study performed in China suggested a benefit in low/intermediate-risk patients from ATRA-ATO containing maintenance; however, there was significant heterogeneity among the induction and consolidation regimens these patients received (73).

As a new recommendation as of their 2019 update to their guidelines in the management of APL, the European LeukemiaNet advises against maintenance in patients with low/intermediate-risk APL treated w/chemotherapy-free (ATRA-based) induction (39). The question remains, however, whether maintenance benefits high-risk patients induced with ATRA/ATO/anthracycline and consolidated with ATRA/ATO who are PML-RARα-negative by PCR at the end of consolidation.

### Relapsed and refractory disease

Relapse, which with modern therapy occurs in less than 5-10% of patients, typically occurs within the first three years of first complete remission (CR1) and rarely later (74). The activity of ATO in relapsed disease has been demonstrated in multiple studies (75–77). The benefit of rechallenge with ATRA has been less clear: a 2003 study showed no benefit to adding ATRA to ATO in relapsed disease, with both arms achieving a CR rate of 80% after one induction cycle (78). This is likely due to intrinsic ATRA resistance present at relapse, with the known occurrence of missense mutations affecting the ATRA-binding domain of the RARα region of the PML-RARα fusion protein (78, 79). The combination of ATO and other agents such as idarubicin and GO have also shown efficacy in regaining molecular CR (77, 80).

Historically, salvage therapy in relapsed AML has consisted of induction of second remission followed by autologous or allogeneic stem cell transplantation (SCT). Due to the reduced tolerability, reduced treatment-related morbidity and mortality, and lack of clear benefit over autologous SCT in CR2, allogeneic SCT is typically reserved for second-and-beyond relapses. Indeed, one retrospective study of 122 relapsed patients demonstrated a 7-year OS and TRM of 59.8% and 6% vs. 51.8% and 39% in autologous vs. allogeneic SCT, respectively, performed in second CR (CR2) (81). SCT High-dose chemotherapy with autologous SCT can cure 50-70% of patients with relapsed APL, and perhaps as high as 87% in those patients who achieve MRD-negative CR2 (complete molecular response) at the time of stem cell collection (81). PCR-positivity for PML-RARα at the time of autologous SCT appears to be a strong predictor of post-transplant relapse (82); allogeneic SCT is therefore a reasonable consideration in those patients unable to reach a complete molecular response ahead of planned transplantation.

Autologous SCT is not without risk, including secondary malignancy associated with high-dose conditioning chemotherapy (81, 82). Hence, an important question is whether some patients in first relapse may be consolidated without transplantation. Although consolidation with autologous SCT consistent shows improved DFS with ATO induction and consolidation alone, it does appear that a significant subset of patients (up to one third) experience long-term DFS with ATO alone (83, 84). Additionally, single agent GO has shown excellent efficacy in regaining molecular remission in singly and multiple refractory APL. In a 2004 study, 13 of 13 patients who received three planned doses of GO at a dose of 6mg/m2 achieved molecular CR; however, 7 responders experienced relapse within 15 months, 2 of whom received GO again and achieved yet another molecular remission (85). Tamibarotene, a novel retinoid with increased differentiating activity compared to ATRA and with a different binding pattern which may circumvent resistance, showed modest activity in a phase II study as a single agent in relapsed APL with a 21% molecular remission rate, albeit with subsequent relapse in 7 of 9 treated patients (86). This agent certainly warrants further study as a combination therapy; in fact, a group in Japan reported the case of a patient with multiple relapses after ATO-based therapy and autologous SCT who achieved molecular remission with tamibarotene and ATO and proceeded to allogeneic SCT (87).

Venetoclax, an oral inhibitor of the anti-apoptotic protein b-cell lymphoma 2 (BCL-2), has shown remarkable efficacy in acute myeloid leukemia in combination with the hypomethylating agents (HMA). *In vitro* studies have demonstrated that APL cell lines are sensitive this agent (88). Case reports have been published exhibiting a CR in relapsed ATRA-resistant APL with the combination of venetoclax and the HMA decitabine (89), as well as the clearance of CNS involvement of APL with single agent venetoclax alongside intrathecal therapy that was resistant to high-dose methotrexate and intermediate-dose cytarabine (90). A clinical trial performed in 2020-2021 studied the efficacy of the V-CAG regimen (venetoclax, cytarabine, aclarubicin, and granulocyte colony-stimulating factor) in nine patients with relapsed or refractory ATRA-resistant APL. Eight of nine patients achieved CR, three of whom achieved a complete molecular response (91). Venetoclax in combination with an HMA or chemotherapy may be considered for disease resistant to ATRA/ATO. Finally, oral arsenic trioxide, discussed in detail further in this review, has shown efficacy in relapsed disease which appears comparable to standard intravenous (IV) ATO.

In summary, ATO-based induction with consolidation either with autologous or allogeneic SCT for patients with MRD negativity or positivity, respectively, remains the recommended treatment for patients in first relapse, with extended ATO consolidation and GO as a single or combination agent remaining a reasonable option for patients who cannot or prefer not to undergo SCT.

### Secondary APL

Therapy-related acute myeloid leukemia (tAML) is, in comparison to *de novo* disease, a high-risk subtype with lower response rates and higher rates of relapse, and for the vast majority of patients is potentially curable only with allogeneic SCT. Like other subtypes of AML, therapy-related APL (t-APL) may occur between months and years after exposure to a topoisomerase II inhibitor or alkylating agent. Indeed, tAPL has also been described in patients receiving single agent mitoxantrone for treatment of multiple sclerosis. Unlike other subtypes of tAML, patients with tAPL appear to experience similarly excellent outcomes as those with *de novo* APL. Therefore, management of tAPL with a risk-adapted approach leads to cure for most patients, and aside from earlier consideration of allogeneic SCT in relapsed disease, management of tAPL is analogous to that of *de novo* disease (92–94).

### Special focus on oral arsenic

Above, we discussed the utilization of novel agents such as GO and tamibarotene in the treatment of APL. The majority of patients, however, will experience excellent outcomes with standard ATRA/ATO based therapy, and a focus on quality of life (QoL) has emerged in light of the high rates of DFS. Many patients may find outpatient administration of IV ATO to be a time-intensive and cumbersome reminder that they are being treated for an aggressive malignancy despite otherwise feeling well, thereby negatively affecting QoL. In recent years, oral formulations of arsenic have been shown to be cost-effective and equivalently bioavailable alternatives to IV ATO (95, 96), and have the potential to transform ATO-based consolidation with a marked improvement in QoL. Oral ATO is commercially available in some parts of the world, including China, where the most experience has taken place with this agent. The oral realgar–indigo naturalis formula (RIF) was launched in China in 2009 and reports show comparable clinical efficacy when compared to the standard IV formulation (including for high-risk patients), with a favorable safety profile (less QTc prolongation and hepatotoxicity when compared to IV ATO) and reduced cost (96, 97). A 2018 phase III randomized control trial in China found oral arsenic (RIF) to be noninferior to IV arsenic in the upfront treatment of non-high-risk APL (98). Next, in 2020, Ravandi and colleagues confirmed the safety and bioavailability of the ORH-2014 oral arsenic formulation (99). Oral ATO has also shown exciting clinical efficacy in relapsed disease (100, 101). We are optimistic that oral ATO will replace the standard IV formulation, improving QoL and reducing costs for patients and the healthcare systems in which they are treated.

## Conclusions

Mutation-targeted therapy has transformed the treatment of APL, and most deaths now occur not from failure to induce or maintain remission but rather from early disease-related mortality. Much progress has been made in the treatment of high-risk patients and those with relapsed disease, and the advent of oral arsenic may significantly improve QoL and reduce healthcare costs for patients receiving consolidation. Certain key questions do remain: Do high-risk patients treated with ATO and anthracyclines derive more benefit than harm, including harm to QoL, from extended maintenance therapy? Which patients require prophylactic intrathecal therapy, given that ATO is believed to be effective in treating the CNS? Can we further stratify high-risk patients to identify those who may be safely treated with a chemotherapy-free approach? The story of the evolution of the treatment is APL is certainly exciting and serves as a model of curative targeted therapy; however, further progress is needed, most urgently in the reduction of induction mortality. Novel therapies, improved education and administration of existing therapies, and the widespread implementation of an oral alternative to IV ATO may further improve the lives of patients affected by APL.

## Author contributions

SI, LE, MS, and JW selected the literature and wrote and reviewed the manuscript. MS designed the figures. All authors contributed to the article and approved the submitted version.
